# Media Exposure to Armed Conflict: Dispositional Optimism and Self-Mastery Moderate Distress and Post-Traumatic Symptoms among Adolescents

**DOI:** 10.3390/ijerph191811216

**Published:** 2022-09-07

**Authors:** Ayelet Pe’er, Michelle Slone

**Affiliations:** 1 School of Psychological Sciences, Tel Aviv University, Tel Aviv 6997801, Israel; 2 Baruch Ivcher School of Psychology, Reichman University, Herzliya 4610101, Israel

**Keywords:** adolescence, armed conflict, media, post-traumatic symptoms, self-mastery, optimism, resilience

## Abstract

Background: Rapid technological developments enable the immediate transmission of armed conflict events through a variety of media channels, inducing mass anxiety, fear, and helplessness. Youth are particularly vulnerable and face new challenges as a result of this exposure. The effects of media exposure to such events on psychological distress and post-traumatic symptoms were examined. Methods: A total of 161 participants aged 13–18 years completed a questionnaire battery that included measures of media exposure to armed conflict events, previous direct exposure to armed conflict events, psychological distress, post-traumatic symptoms, dispositional optimism, and self-mastery. A structural equation model (SEM) approach was employed for data analysis. Results: The extent of media exposure to armed conflict was directly associated with psychological distress and post-traumatic symptoms. Dispositional optimism moderated the association between media exposure and psychological distress, while self-mastery moderated the association between media exposure and post-traumatic symptoms. The effects of the Internet factor of media exposure, which included social media, were particularly disturbing as neither of the resilience factors moderated negative outcomes. Conclusions: The findings suggest that clinical interventions to enhance dispositional optimism and self-mastery as well as other potential resilience factors can protect adolescents from the severe effects of media exposure to violent armed conflict events. Developmental and public health implications related to vulnerabilities and resilience during adolescence are discussed.

## 1. Introduction

Social media have become a central focus in the life of adolescents [1], with reports showing that 97% of adolescents are active on social media today [2]. Studies of the emotional effects of social media usage have shown associations with both positive and negative developmental aspects such as self-esteem, social connectedness, internalizing problems, risk-taking behaviors, and sexual self-exploration [3,4,5,6,7]. Therefore, the impact of social media on adolescent well-being has become a global priority [8]. This focus is most relevant in the context of adolescents’ media exposure to war and armed conflict events, since the materials available in new technological platforms are open to the immediate online distribution of the virtually unlimited content of abhorrent violent scenes of these conflict events from all over the world.

In addition, the increasing incidence of war, armed conflict, and terrorism has become a global problem, reaching deep into societies and communities, targeting civilians and inducing mass anxiety, fear, a continuous sense of threat, and helplessness [9,10,11]. Millions of children and adolescents are indirectly exposed to war, armed conflict, and terrorism through the media, with adverse effects [12,13,14,15]. The use of smartphones and computers in the hands of the youth may be portals to extremely dangerous places. Violent scenes of armed conflict can be projected into the immediate environment, producing a new dimension of experience of the world [16]. The scenes are uncensored and their consumption may be unmonitored or unsupervised by responsible adults. In the case of adolescents, the effects of this new dimension of experience can overlap with the complexities of the developmental period. In light of these complex combinations, the present study examined the exposure of adolescents to armed conflict events through different media channels, the psychological effects of this exposure, and personal factors that could moderate negative effects.

### 1.1. Adolescents in a Changing World of Media 

Among the population groups affected by war and terrorism, the youth are particularly vulnerable [17]. The developmental challenges of childhood and adolescence may become more difficult when growing up in insecure and unstable environments, which often undermine assumptions about the world as a safe place [18]. These challenges include the consolidation of self, sense of identity, regulation of impulses, and striving for self-autonomy [19]. Adolescence is frequently characterized by adventure-seeking, sensation-seeking, and risk-taking behavior [20], which can produce greater vulnerability. Furthermore, in armed conflict areas, media exposure to violent armed conflict events, and often terrorism, is almost impossible to avoid [13]. One of the main characteristics of adolescents’ media exposure to terrorism in such environments is the accumulation of multiple and frequent exposure to violent and shocking materials, rather than an acute single exposure [21]. 

Adolescents can be deeply affected by the images viewed across the various media platforms following armed conflict events and acts of terrorism. Studies have shown that realistic violence has greater effects on children and adolescents than fictional violence [22,23]. Moreover, among the different types of realistic violence portrayed via the media, armed conflict has been found to be most stressful for adolescents [24]. This is possibly due to the actual real-life danger and sense of threat embedded in the media exposure to armed conflict where there is a need to assess the actual threat [25]. 

The effects of viewing these frightening scenes range across symptoms of anxiety, sadness, isolation, and general psychological distress [15,26,27] to elevated perceptions of personal risk and disrupted daily routines and activities [24,28]. Furthermore, media exposure to terrorism and armed conflict has been found to predict a wide range of functional outcomes in children and adolescents following terrorist attacks including PTS symptoms, aggression [21], sleep disorders, behavioral withdrawal [29,30,31], and the radicalization of ideological beliefs [32].

Research suggests that for adolescents, the frequency, duration, and type of media exposure predict the psychological effects of media exposure to armed conflict events. For example, greater media exposure, either in duration measured as longer hours or intensity reflected in highly gruesome images, has been associated with a higher prevalence of distress symptoms [21,26,33]. Most studies have focused on television viewing of war, armed conflict, and terrorism, with little focus on other media platforms such as the Internet and social media. Television was previously rated by youth as the most extensively used medium when compared to the radio, Internet, text messages, and newspapers [34]. This is no longer the case and warrants the examination of the effects on youth of the consumption of armed conflict events via more modern and current media platforms. The present study examined the extent of exposure to three categories of media exposure to armed conflict events: conventional media such as in television, radio, and newspapers; Internet media as in social media and websites; and video viewing as in YouTube and WhatsApp. The differential effect of the three categories in the extent of exposure on psychological distress and post-traumatic symptoms was also examined. 

### 1.2. Terrorism, Armed Conflict, and the Mass Media 

Social media and other Internet platforms have been embraced by military organizations and terrorists for the same reasons as they have been engaged by other organizations, namely, their capacity to expand their reach and influence and their efficiency in the dissemination of propaganda [35]. Media coverage can evoke attention and emotions and dramatize contents [36,37], with the exaggeration of factual content and elevated threat perception [36,38,39]. The power of mass media in influencing public perception often leads to stereotyping, trivialization, misinformation, and exaggeration [40]. Many terrorist and military groups have come to recognize that the Internet is a powerful tool that can be deliberately and tactically used to advance their strategic objectives of online propaganda [41]. 

The feasibility of uploading and publishing any information, with no need for monitoring or approval from authorities, is an immense advantage [14,16,41,42]. Moreover, social media platforms such as Facebook, Twitter, Instagram, or Tik-Tok enable the creation of social networks [43]. The digitalization of Internet information allows for infinite copying and spreading of material via social media. Many radical groups use these advantages of the Internet and social media to optimize reaching out to as many people all over the world as possible, making the Internet a powerful catalyst for facilitating terrorist and military operations [42]. 

In the case of terrorist groups, the intentional use of the media has been given the term “*theater of terror*”, an expression that is extremely relevant today [44] and refers to the dramatic use of the media in the attempt to exhibit their activities on the global stage. Terrorists frequently choreograph a “show of terrorism” on mass media with the objective of presenting a dramatic spectacle to capture media attention [45,46,47]. Widespread exposure to armed conflict events, war, and terrorism through mass media has enabled a magnification of their impact and the gaining of global attention [48,49]. A clear example of this phenomenon can be seen in the ISIS videos of atrocities, which rapidly spread worldwide via social media [41]. 

The authors have pointed out the interdependence, or even symbiotic relations, between the media and terrorism and other military activities [50,51]. Terrorists frequently exploit the media to achieve political recognition, present their cause, transmit messages, and induce fear in the general public whereas the media exploits terrorism to provide the public with up-to-date news. Furthermore, since terrorist acts are by their very nature unexpected, dramatic, exceptionally violent and distressing, they are perceived by the media as sought-after news items, deserving of extensive coverage [50]. 

### 1.3. Resilience Factors–Optimism and Self-Mastery

With regard to terrorism and armed conflict, a wide array of post-exposure consequences of exposure has been documented for youth. Pre-disaster factors such as previous exposure to war and armed conflict may increase vulnerability [52]. Alternately, individual variations in traits could moderate responses to terrorism and armed conflict. Such factors include personal coping strategies [53,54,55,56], social support [57], well-being [58], attribution of meaning [59,60], sense of coherence [54,61,62], and self-complexity [63]. Two of the widely reported resilience factors in the face of traumatic exposure are dispositional optimism [43,64,65,66,67] and self-mastery [68,69,70,71,72,73]. While these factors were found to moderate the negative effects of direct exposure to trauma, in the present study, dispositional optimism and self-mastery were examined as potential resilience factors for adolescents exposed to armed conflict and terrorism via the media. 

#### 1.3.1. Self-Mastery

Self-mastery and similar concepts such as self-efficacy refer to the belief in the competence and personal capacity to solve problems and take actions that are necessary to manage life situations [74]. The concept reflects the extent to which people perceive having control over their lives as well as an internal feeling of strength and the capacity to cope with and overcome obstacles [75,76]. High self-mastery has been repeatedly associated with lower levels of psychological distress and psychiatric symptoms [68,69,70,77,78] including post-traumatic symptoms [68,73]. Self-mastery has been shown to be a personality characteristic that moderates negative effects and improves prognosis in the aftermath of various traumatic events [69,70,72,75,77,79,80]. 

In the context of terrorism and armed conflict, self-mastery acts as a resilience factor [71,72] that may even be related to post-traumatic growth [69]. In a recent study on exposure to terrorism, self-mastery had both a direct negative association with psychological symptoms and a mediation effect between exposure to life threatening events, and psychological symptoms [72]. However, traumatic exposure such as exposure to terrorism and armed conflict may also lead to a decrease in levels of self-mastery [68,81].

#### 1.3.2. Dispositional Optimism

Dispositional optimism is often defined as the extent to which people hold a general expectation that good outcomes will occur in the face of adversity [82,83] or that their future will be generally positive [84]. Optimism is a form of explanation or interpretation of life events as good for the individual [85,86]. Research has indicated the importance of dispositional optimism as a key resource in times of extreme stress and trauma [87,88] including terrorism exposure [43,84,89]. 

The findings are less conclusive among adolescents. War and conflict produce chaotic and threatening environments that make it difficult for adolescents to maintain a sense of control or a sense of self-mastery. A relatively recent study of direct exposure to armed conflict events found that an intervention for the enhancement of self-mastery was effective in reducing psychological distress and emotional symptoms among adolescents, while the control group participants reported increases in these indices [71]. The present study aimed to examine the moderating effects of self-mastery and dispositional optimism in the relation between media exposure to armed conflict and terrorism and psychological outcomes among adolescents. 

### 1.4. The Israeli Context

The long-standing violent and complex Israeli–Palestinian conflict has caused a pain-fully large number of casualties and fatalities on both sides [52,90,91]. The Israeli–Palestinian conflict has been a media focus with extensive coverage on central television channels, social media, Internet sites and mobile news applications, spreading the distressing scenes to the public locally and globally [14,34]. In addition, the intentional use of the media by both sides has increased the visibility of the long-lasting conflict [41,48]. In May 2021, tensions between the Israelis and the Palestinians reached yet another peak of violence. After several weeks of escalating tensions and hostilities, on 10 May 2021, the situation exploded into a military stand-off with rockets fired from Gaza onto centers of civilian populations in Israel, and Israel declaring a military operation in Gaza named “Operation Guardian of the Walls”. The operation lasted 12 days and was a violent, difficult, and complicated period for both the Israelis and the Palestinians. Unfortunately, we were only able to examine the effects on the Israeli population. Quite understandably, both populations experienced much suffering and distress and sought cognitive clarifications of the unfolding events. The present study was conducted immediately after “Operation Guardian of the Walls” to examine the possible effects of media exposure to the armed conflict events that had occurred on the psychological distress and post-traumatic symptoms of Israeli adolescents. 

### 1.5. Hypotheses

In light of the rationale above, this study proposed three hypotheses and one exploratory question.

The first hypothesis states that the extent of media exposure regarding the occurrence of armed conflict events, operationalized as the extent of exposure to different types of media, will be directly associated with psychological distress and post-traumatic symptoms. 

The second hypothesis states that the severity of direct exposure to actual armed conflict events will be directly associated with psychological distress and post-traumatic symptoms.

The third hypothesis predicts that self-mastery and dispositional optimism will moderate the association between the exposure variables of the extent of media exposure to armed conflict events and the severity of direct exposure to armed conflict events and the outcome variables of psychological distress and post-traumatic symptoms.

The exploratory question related to the possible differentiated effects of different categories of media platforms included in the general media exposure variable. The moderating role of self-mastery and dispositional optimism for different categories of media exposure to armed conflict events on psychological distress and post-traumatic symptoms was examined, for which a directional hypothesis could not be formulated. 

## 2. Materials and Methods

### 2.1. Participants

The sample consisted of 161 participants in the age range 12.5–18.7 (mean age = 15.82, SD = 1.57). Sixty four percent of the sample were females. Eighty five percent of the sample attended a regular school, 1.2% attended a special education school, and 13.2% “other”. Demographic data indicated that the sample consisted of adolescents from across the country and that socioeconomic status varied from low to moderate to high. The recruitment of participants during the sensitive and distressing period had its limitations. While over 160 adolescents were recruited after parental consent, representative in terms of gender and age, we were only able to reach Jewish schools and therefore the sample is not representative of the Arab population in Israel. 

No significant associations were found between any of the socio-demographic variables and the dependent variables (psychological distress and post-traumatic symptoms), except for gender. Female adolescents reported significantly higher psychological distress (M = 2.45, SD = 0.87) and post-traumatic symptom scores (M = 1.55, SD = 0.66) than reports of male participants on the levels of psychological distress (M = 2.08, SD = 0.78) and post-traumatic symptoms (M = 1.24, SD = 0.41) (t (160) = 2.66, *p* = 0.01; t (160) = 3.18, *p* = 0.002, respectively).

### 2.2. Instruments

#### 2.2.1. Media Exposure to Armed Conflict Events

Media exposure to armed conflict events in the tense period leading up to and during the military operation was assessed using an exposure scale constructed for the purpose of this study. The study was conducted immediately at the end of the military operation, which lasted for 12 days. The period of escalating hostilities in the region and the two-week military operation produced a highly tense and violent time in the region for both sides, with many armed conflict events. Participants were asked to indicate the extent to which they used different media platforms regarding the armed conflict events that had occurred, for example, after a barrage of missiles or a terror attack in a city center. The participants were presented with 10 different media platforms that were rated for usage after the occurrence of these events—Facebook, Instagram, WhatsApp, newspapers, radio, television, phone alerts, YouTube, web, and other. Responses were rated on a scale ranging from 1 (not at all) to 5 (all the time). The total media exposure score was calculated by averaging the items, with a higher score representing a greater total media exposure of armed conflict events. As this measure was constructed for the present study, no previous psychometric properties have been documented. The internal reliability of the scale in this study was α = 0.66. 

For the exploratory question, ratings of the 10 media platforms were analyzed to examine the possible different effects of the usage of different categories of media consumption on the study variables. In this analysis, the extent of media consumption of each of these categories was examined. 

#### 2.2.2. Dispositional Optimism

Dispositional optimism was assessed using the Life Orientation Test–Revised (LOT–R), [92]. The LOT–R consists of 10 items composed of six relevant items (e.g., “In uncertain times, I usually expect the best”) and four irrelevant items (e.g., “It is easy for me to relax”). Participants were requested to rate their agreement with each item on a scale from 0 (strongly disagree) to 4 (strongly agree). The level of dispositional optimism was calculated as the average score of the six relevant items with high scores indicating high levels of dispositional optimism. LOT–R has demonstrated adequate psychometric properties in past research [92] and has shown acceptable internal reliability levels (α = 0.78). The Cronbach reliability in the current study was α = 0.79.

#### 2.2.3. Self-Mastery

Self-mastery was measured using the Mastery Scale [93], which reflects the general perceived control. The scale includes seven items rated on a seven-point scale from 1 (not at all) to 7 (very much), for example, “What happens to me in the future mostly depends on me” or “I can do just about anything I really set my mind to do”. The final score was computed by averaging the relevant items. The scale reports the test–retest reliability of 0.85 or above and good internal reliability levels (α = 0.75) [94]. In the present study, the internal reliability was α = 0.80.

#### 2.2.4. Psychological Distress

Psychological distress was measured by means of the Brief Symptom Inventory-18 [95], which has been found to be an effective screen for psychological distress and psychiatric disorders. The questionnaire consists of 18 self-reported items rated for the frequency of symptoms experienced over the past month from 0 (not at all) to 4 (very much) such as “feeling hopeless about the future”, “nervousness or shakiness inside”, or “feeling so restless you couldn’t sit still”. The BSI-18 yields three subscale scores—depression, anxiety, and somatization, and a Global Severity Score (GSI) calculated as the average of all items. The GSI is considered the best indicator of the depth of distress [95]. Internal consistencies of the BSI-18 range from 0.74 to 0.89 [96]. In the current study, a high Cronbach reliability was found (α = 0.92).

#### 2.2.5. Post-Traumatic Symptoms

Post-traumatic symptoms were measured with the PTSD Symptom Inventory [97], which consists of 17 items corresponding to the Diagnostic and Statistical Manual of Mental Disorders PTSD symptoms (DSM–IV 4th ed.; [98]). Items were rated on a scale of 0–3 for combined frequency and severity of symptoms in the past 2 weeks (0 = not at all, 1 = once per week or less, 2 = 2–4 times per week, or 3 = 5 or more times per week) and include statements such as “having recurrent bad dreams or nightmares about the trauma” or “feeling detached or cut off from others around you since the trauma”. Ratings were averaged to a general score with higher scores reflecting more post-traumatic symptoms. The scale has been shown to have high internal consistency (α = 0.85) and excellent inter-rater reliability for the overall severity of symptoms (*r* = 0.97), [97], and has been widely used in the context of terrorism, armed conflict, and other traumas [99]. Internal consistency in the current study was high (α = 0.95).

#### 2.2.6. Previous Exposure to Direct Armed Conflict Events

The severity of prior exposure to direct armed conflict events was measured using the Political Life Events Scale (PLE; [100]). Participants were asked to mark their exposure to 20 different armed conflict related events. The severity of exposure score was computed as the sum of weighted scores assigned to each experienced event according to the formula 1 = low severity events (e.g., “I was present in a situation where there was a suspected dangerous explosive”); 2 = moderate severity events (e.g., “My property was harmed as a result of terrorism”); 3 = high severity events (e.g., “ Exposure to gunshot or the use of other weapons or explosives “) [63,90]. The PLE scale has been widely used and validity studies have yielded excellent results [90]. Since there is no rationale for the assumption of a relation between exposure to the discrete items, internal consistency was not calculated for this scale.

### 2.3. Procedure

After receiving authorization from the Tel-Aviv University Ethics Committee and both parental and participant informed consent, all participants completed the questionnaire battery via the Internet using the Qualtrics web-survey software, which allowed for anonymity and confidentiality. Participants were informed that they could terminate their participation at any point. 

## 3. Results

The means, standard deviations, and bivariate correlations of the study variables are presented in Table 1.

Simple correlations indicated that psychological distress and post-traumatic symptoms were positively moderately associated, indicative of a unique contribution of each psychological outcome. Both psychological distress and post-traumatic symptoms were positively associated with media exposure to armed conflict events.

Additionally, both psychological distress and post-traumatic symptoms were negatively associated with dispositional optimism and self-mastery. Post-traumatic symptoms were also positively associated with previous direct exposure to armed conflict events. Finally, dispositional optimism was positively related to self-mastery and negatively related to media exposure to armed conflict events. 

**Figure 1:**
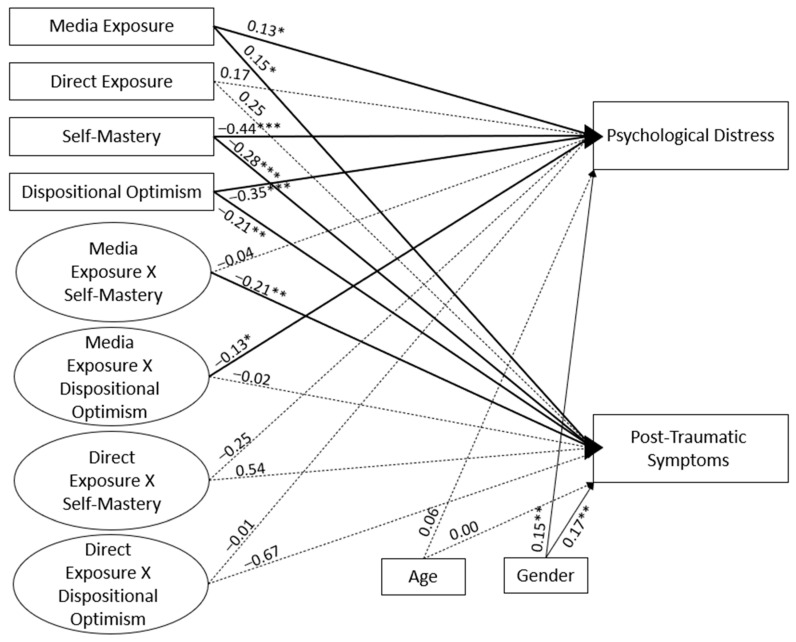
The structural equation model to explain the variance in general distress and post-traumatic symptoms by media exposure, previous direct exposure, self-mastery, and dispositional optimism. * *p* < 0.05; ** *p* < 0.01; *** *p* < 0.001. Coefficients are standardized.

In order to examine whether media exposure and previous direct armed conflict event exposure predicted both psychological distress and post-traumatic symptoms as a function of dispositional optimism and self-mastery, a structural equation model (SEM) approach was employed using AMOS 4 with the maximum likelihood estimation. The model controlled for age and gender. Overall, the model demonstrated good fit [χ2(13) = 15.37, *p* = 0.569, CFI = 1.00, TLI = 1.00, RMSEA = 0.000], see Figure 1.

As presented in Figure 1, the first hypothesis regarding the relation between media exposure to armed conflict events and psychological distress (β = 0.13, *p* = 0.012) and post-traumatic symptoms was confirmed (β = 0.15, *p* = 0.023). In addition, both psychological distress and post-traumatic symptoms were predicted by self-mastery (β = −0.44, *p* < 0.001 and β = −0.28, *p* < 0.001, respectively) and dispositional optimism (β = −0.35, *p* < 0.001 and β = −0.21, *p* = 0.005, respectively). The second hypothesis that predicted a relation between previous direct exposure to armed conflict events and negative psychological outcomes was not confirmed, as post-traumatic symptoms (β = 0.25, *p* = 0.529) and psychological distress (β = 0.17, *p* > 0.05) were not predicted by greater previous direct exposure. 

The third hypothesis that related to the moderating effects of self-mastery and dispositional optimism revealed a differential pattern. Self-mastery moderated the effect of media exposure to armed conflict events on the post-traumatic symptoms. Disposition optimism, on the other hand, moderated the effect of media exposure to armed conflict events on psychological distress. To illustrate these interactions, the sample was split into two halves by the median value of self-mastery score for the first interaction, and split into two halves by the median value of the disposition optimism score for the second interaction. The model described in Figure 1 was then conducted for each half of the sample, without the interaction terms. Analyses of the simple slopes for the interaction between media exposure and self-mastery in predicting post-traumatic symptoms revealed that the association between media exposure and post-traumatic symptoms was significant for low levels of self-mastery, *Beta =* 0.19, *p* = 0.032, but not for high levels, *Beta =* −0.05, *p* = 0.621. For the interaction between media exposure and dispositional optimism in predicting psychological distress, analyses of the simple slopes revealed that the association between media exposure and psychological distress was significant only for low levels of dispositional optimism, *Beta =* 0.25, *p <* 0.001, but not for high levels, *Beta =* 0.18, *p* = 0.089. 

The exploratory question related to the potential differentiated effect of different categories of media exposure to armed conflict events, which comprised the media exposure score presented in Figure 1. This analysis examined the moderating role of self-mastery and dispositional optimism as potential resilience factors in the relation between the extent of exposure to each of the different categories of media exposure and psychological distress and post-traumatic symptoms. For this purpose, factor analysis was conducted on the total media exposure variable and then the same model was re-examined, but this time with the factors of media exposure yielded by the factor analysis, instead of the total score. An exploratory factor analysis with Varimax rotation on the media exposure items revealed three factors explaining 60.18% of the variance. The factors were interpreted as representing ‘conventional’ media (newspapers, radio, and television news channels), ‘Internet’ media (social media, Internet news channels, and phone alerts), and ‘video’ media (WhatsApp and YouTube), and were moderately correlated (correlations ranged from *r* = 0.17 to *r* = 0.31). The same path model was re-examined with the three factors of media exposure and the interaction between each media exposure factor with dispositional optimism and self-mastery on psychological distress and post-traumatic symptoms. 

Results of the path model again indicated a good fit [χ2(25) = 31.44, *p* = 0.175, CFI = 0.99, TLI = 0.95, RMSEA = 0.04]. As in the previous model, both psychological distress and post-traumatic symptoms were predicted by self-mastery and dispositional optimism. In addition, the extent of use of Internet media exposure predicted psychological distress (*Beta* = 0.12, *p* = 0.035), and the extent of use of video media exposure predicted post-traumatic symptoms (*Beta* = 0.14, *p* = 0.038). Finally, two significant interactions were found. First, an interaction effect of *video* media exposure and self-mastery on post-traumatic symptoms was found (*Beta* = −0.24, *p <* 0.001). In addition, an interaction between *conventional* media exposure and dispositional optimism in predicting post-traumatic symptoms emerged (*Beta* = 0.21, *p* = 0.012). Simple slope analyses indicated that the extent of media exposure of both video and conventional platforms was associated with more severe post-traumatic symptoms, but only for low levels of self-mastery and dispositional optimism, respectively (*Beta* = 0.27, *p* = 0.004; *Beta* = 0.45, *p* = 0.003, respectively), and not for high levels (*Beta* = 0.03, *p* = 0.823; *Beta =* −0.07, *p* = 0.448, respectively)

## 4. Discussion

This study examined the effects of adolescent media exposure to armed conflict events on post-traumatic symptoms and psychological distress as well as possible interactive factors including previous direct armed conflict event exposure, self-mastery, and dispositional optimism. The results showed that the extent of media exposure to armed conflict was directly associated with psychological distress and post-traumatic symptoms. Previous direct exposure to armed conflict events was not associated with post-traumatic symptoms or with psychological distress. In addition, dispositional optimism moderated the association between the extent of general media exposure to armed conflict events and psychological distress, while self-mastery moderated the association between the extent of general media exposure to armed conflict events and post-traumatic symptoms. The exploratory question related to the moderating role of self-mastery and dispositional optimism for the extent of the usage of each of the different categories of media exposure on psychological distress and post-traumatic symptoms. 

The first hypothesis stated that the extent of general media exposure to armed conflict events, operationalized as the extent of usage of all types of media, will be directly related to psychological distress and post-traumatic symptoms. This hypothesis was confirmed. In addition, a moderate correlation was found between psychological distress and post-traumatic symptoms, indicating that each made a unique contribution to the exposure outcomes. This indicates that the exposure outcomes can present as post-traumatic symptoms or as more specific symptoms of anxiety, depression, and somatization, as measured by the BSI. These findings are congruent with previous studies [21,101] and highlight the power of the media as the means through which armed conflict events and terrorism spread fear and threat in communities and among individuals that are far away from the specific site of the attack. The findings of this study suggest that the effects of media exposure of these horrifying scenes are associated with severe psychological distress and post-traumatic symptoms. Adolescents, with their proficient and frequent use of different types of media, have become a default target for exposure to this material.

The second hypothesis, which stated that the severity of previous direct exposure to armed conflict events will be directly associated with psychological distress and post-traumatic symptoms, was not confirmed. Correlations between the previous direct armed conflict exposure and post-traumatic symptoms and psychological distress were not significant. This is a surprising finding considering the widely documented relation between the exposure to terrorism and armed conflict events and post-traumatic symptoms [21,34,101,102,103]. However, studies have shown that the relation between direct exposure to armed conflict and psychological distress can often be weak or even non-linear [52,104,105] such as the finding of an inverted U shaped curve in the relation between exposure and psychological outcomes [106]. A possible reason for this is the finding of moderating or mediating factors that can intervene in the relation between direct exposure and outcomes [55,56,57,59,64]. It is possible that in our model, the effect of direct exposure is moderated by factors not included in this study. Alternately, the relation between direct exposure and outcomes was overshadowed by the robust effect of media exposure, which was significantly related to both outcome measures—psychological distress and post-traumatic symptoms. It seems that the inclusion of both indirect media exposure as well as direct exposure to armed conflict events in the model revealed the far-reaching effect of media exposure, even when controlling for direct exposure. It could be that individuals who experienced the most severe type of personal direct exposure were less likely and unamenable for participation in research. It is also possible that this finding reflects the dramatization, exaggeration, and amplification by the media, extending its collective impact [36,39,40], as opposed to personal events that are not exhibited and magnified on the global stage [44,48,49]. Social media have currently become one of the most substantial channels to influence public opinion in developed societies [40]. It seems that the inclusion of both indirect media exposure as well as direct exposure to armed conflict events in the model revealed the far-reaching effect of media exposure, even when controlling for direct exposure. These intrinsic relations should be further examined in future studies. 

The third hypothesis predicted that self-mastery and dispositional optimism will moderate the association between the exposure variables (i.e., extent of general media exposure to armed conflict events and severity of direct exposure to armed conflict events) and the outcome variables of psychological distress and post-traumatic symptoms. This hypothesis was partially confirmed. A significant negative correlation was found between dispositional optimism and self-mastery and psychological distress and post-traumatic symptoms. This finding corroborates the existing research literature [43,64,65,66,67,71,72] and provides further validity for the measurement of these variables in the present study. 

The significant association between the extent of general media exposure to armed conflict events and psychological distress was only found among participants with low levels of dispositional optimism and not among participants with high levels of dispositional optimism. This finding suggests that dispositional optimism acts as a resilience factor in mitigating general psychological distress, which presents as anxiety, depression, and somatization after media exposure to armed conflict events. 

Additionally, a significant association between the extent of general media exposure to armed conflict events and post-traumatic symptoms was found only among the participants with low levels of self-mastery and not among the participants with high levels of self-mastery, suggesting that self-mastery acts as a resilience factor in the association between media exposure and post-traumatic symptoms. The moderating function of dispositional optimism and self-mastery has been well-established [34,43,65,66,69,71,72]. The differential moderating effects of dispositional optimism and self-mastery is not easily interpretable and requires further study. In the present study, dispositional optimism moderated the effects of media exposure on psychological distress, while self-mastery moderated the effects of media exposure on post-traumatic symptoms. Possibly, a strong sense of agency and self-belief in the ability to handle difficult situations and to cope with adversity counters some of the symptoms characterizing post-traumatic stress, particularly, avoidance and numbing. It is also probable that dispositional optimism produces a more general sense of well-being and better mood, which together reduce the general symptoms of anxiety, depression, somatization, and general distress. It should be noted, however, that these aspects of dispositional optimism do not provide an active coping strategy against the more severe and specific symptoms of trauma.

The finding that dispositional optimism and self-mastery function as resilience factors against media exposure to armed conflict events has not been shown previously. It is important to note that this finding pertains to the media exposure of armed conflict events and terrorism among adolescents. With the advent of different, easily accessible media platforms, notorious for their amenability, adolescents have become particularly avid consumers of the media coverage of war, armed conflict, and terrorism events [21,34,107]. Thus, the sophisticated and efficient use of the media places adolescents under particular risk for negative psychological outcomes. The understanding that dispositional optimism and self-mastery can act as resilience factors that moderate these negative effects has important clinical implications, as these characteristics can be encouraged or enhanced in educational and clinical settings.

The exploratory question examined the moderating role of self-mastery and dispositional optimism for the extent of usage of each of the different categories of media exposure to armed conflict events on psychological distress and post-traumatic symptoms, for which a directional hypothesis could not be formulated. Three categories of media exposure emerged in this analysis—Internet media exposure, conventional media exposure, and video media exposure. The Internet factor of media exposure, which included social media, Internet news channels, and phone alerts, showed a direct association with psychological distress levels, which presented as anxiety, depression, somatization, and general distress. This finding is particularly disturbing given that the Internet has become a major means of media distribution among adolescents, especially via social media platforms such as Facebook, Instagram, Twitter, Tik-Tok, etc. Youth can be exposed to uncensored and highly troubling scenes that are associated with increases in psychological distress including, anxiety, depression, and somatization. These findings emphasize the need to direct public attention to the dire effects to adolescents of uncensored, disturbing, and easily available materials. This study also showed that dispositional optimism and self-mastery did not moderate the association between Internet exposure to armed conflict events and psychological distress and post-traumatic symptoms. Hopefully, there are other variables that moderate the association between Internet exposure to war, armed conflict and terrorism events, and psychological distress. Other such variables should be explored in future research, considering the impact such variables can have on future interventions. 

Even though self-mastery did not moderate the negative effects of Internet media exposure, it was found to moderate the impact of both conventional and video media exposure on post-traumatic symptoms. Dispositional optimism, on the other hand, only moderated the effects of conventional media exposure on general psychological distress, as reflected in the GSI.

### Study Limitations

Despite the aforementioned findings that suggest the complex effects of different types of media exposure and resilience factors on psychological symptoms and post-traumatic symptoms, this study has several limitations that should be addressed in further research. First, this study was based on questionnaires completed on the Internet and therefore may have been susceptible to response bias. Second, this study made use of single-agent, self-reported questionnaires, which would be better complemented with multi-agent reports or behavioral measures. Third, the media exposure to the armed conflict variable was difficult to operationalize, making it necessary to categorize the many platforms of media exposure to armed conflict (e.g., television, social media platforms, and internet news sites) and create measures of usage and the extent of consumption of each of these different platforms. Therefore, this variable was then measured as the sum of consumption of each of the listed platforms. This measurement was unable to distinguish between the extensive consumption of a few favored platforms versus the light consumption of many different platforms. Finally, the sample was comprised of Jewish adolescents, and therefore did not represent the Arab population in Israel. Attention to these limitations in future studies would increase the generalization of these findings.

## 5. Conclusions

The present study advances many suggestions for public policy regarding the mental health of adolescents. The developmental implications relate to our comprehension of the vulnerabilities and resilience during the period of adolescence. Adolescents are commonly exposed to the rapid media distribution of violent scenes, war, armed conflict events, and terrorist acts, and thus are particularly susceptible to significant negative psychological effects. The period of adolescence is characterized by the search for autonomy and the tendency to seek new and adventurous experiences, which could eventuate in high risk-taking behaviors. This, alongside the adolescents’ proficiency in exploiting new media platforms, accessibility to violent materials, and immaturity to cope with these disturbing stimuli, could produce a potent combination that heightens their vulnerability to psychological symptoms. 

Parents are a pivotal factor in supporting an adolescent’s mental health, particularly in an insecure and threatening environment. Thus, public health stakeholders should address ways to inform parents of the potential harmful effects of uncensored and unsupervised media exposure to war, armed conflict, and terrorism events as well as the resilience factors that can mitigate negative outcomes and strategies for reducing exposure. Education and counseling for parents are essential, since the monitoring of adolescents is particularly difficult due to the complexities of the developmental period including the high value placed on protecting privacy and autonomy [108]. 

The present study also provides specific findings regarding the role of dispositional optimism and self-mastery as moderators of the negative effects of media exposure to armed conflict. The findings showed that dispositional optimism moderates psychological distress and that self-mastery moderates post-traumatic symptoms. These findings suggest the possibility of clinically manipulating the levels of dispositional optimism and self-mastery. However, it is important to identify the specific media platforms for which these resilience factors serve as moderators. Despite the partial effectivity of these particular moderating factors, the findings of this study hold promise for the detection of additional resilience factors that could inform the development of interventions for mitigating the negatives effects of media exposure to armed conflict events and terrorism among adolescents.

On a broader level, this study has important consequences for the development and examination of the efficacy of intervention programs to inform, monitor, and treat youth exposed through the media to shocking and violent war, armed conflict, and terrorism events. As several screening and intervention programs have been developed for children exposed to the Israeli–Palestinian conflict in particular [109,110], the identification of risk groups may need to be modified in light of the current findings. There is a need for the development and implementation of developmentally-sensitive community programs to address the difficulties of large populations of children in their natural settings in a cost effective manner. 

Public policy implications suggest that authorities and media stakeholders should consider the trade-off between freedom of information and the adverse effects of media exposure to war, armed conflict, and terrorism on the mental health of the youth. It could be useful to alert new social media propagators of the adverse effects of this uncensored material, which may encourage the development of appropriate strategies to monitor and limit exposure.

This study highlights the complex ethical and regulatory dilemmas involved in relations between the media distribution of violent armed conflict related material and public mental health. This relation has become even more complicated with the development of diverse and ever-increasing social media platforms with which today’s youth are extremely proficient. Mental health practitioners should be informed about the effects and risks to youth, leading to careful consideration of the strategies to limit negative outcomes.

## Figures and Tables

**Table 1:** The means, standard deviations, and bivariate correlations of the study variables (N = 161).

	M	SD	1	2	3	4	5	6
1. Psychological distress	2.32	0.86	1					
2. Post-traumatic symptoms	1.43	0.59	0.65 ***	1				
3. Dispositional optimism	3.28	0.79	−0.62 ***	−0.41 ***	1			
4. Self-mastery	3.66	0.82	−0.65 ***	−0.46 ***	0.53 ***	1		
5. Direct exposure	8.19	4.57	−0.03	0.17 *	−0.04	−0.04	1	
6. Media Exposure	2.35	0.67	0.22 **	0.19 *	−0.17 *	−0.03	−0.01	1

* *p* < 0.05; ** *p* < 0.01; *** *p* < 0.001.

## Data Availability

The data presented in this study are openly available in Mendeley Data at 10.17632/xrthvhmdf9.1.
